# Engineering inclusion bodies for non denaturing extraction of functional proteins

**DOI:** 10.1186/1475-2859-7-34

**Published:** 2008-12-01

**Authors:** Špela Peternel, Joze Grdadolnik, Vladka Gaberc-Porekar, Radovan Komel

**Affiliations:** 1 Department of Biosynthesis and Biotransformation, National Institute of Chemistry, Ljubljana, Slovenia; 2 Department of Molecular Modeling and NMR Spectroscopy, National Institute of Chemistry, Ljubljana, Slovenia

## Abstract

**Background:**

For a long time IBs were considered to be inactive deposits of accumulated target proteins. In our previous studies, we discovered IBs containing a high percentage of correctly folded protein that can be extracted under non-denaturing conditions in biologically active form without applying any renaturation steps. In order to widen the concept of correctly folded protein inside IBs, G-CSF (granulocyte colony stimulating factor) and three additional proteins were chosen for this study: GFP (Green fluorescent protein), His7dN6TNF-α (Truncated form of Tumor necrosis factor α with an N-terminal histidine tag) and dN19 LT-α (Truncated form of Lymphotoxin α).

**Results:**

Four structurally different proteins that accumulate in the bacterial cell in the form of IBs were studied, revealing that distribution of each target protein between the soluble fraction (cytoplasm) and insoluble fraction (IBs) depends on the nature of the target protein.

Irrespective of the folding pattern of each protein, spectroscopy studies have shown that proteins in IBs exhibit similar structural characteristics to the biologically active pure protein when produced at low temperature. In the case of the three studied proteins, G-CSF, His7ΔN6TNF-α, and GFP, a significant amount of protein could be extracted from IBs with 0.2% N-lauroyl sarcosine (NLS) and the proteins retained biological activity although no renaturation procedure was applied.

**Conclusion:**

This study shows that the presence of biologically active proteins inside IBs is more general than usually believed. A large amount of properly folded protein is trapped inside IBs prepared at lower temperatures. This protein can be released from IBs with mild detergents under non-denaturing conditions. Therefore, the active protein can be obtained from such IBs without any renaturation procedure. This is of great importance for the biopharmaceutical industry. Furthermore, such IBs composed of active proteins could also be used as pure nanoparticles in diagnostics, as biocatalysts in enzymatic processes, or even as biopharmaceuticals.

## Background

The formation of inclusion bodies (IBs) upon overexpression of heterologous proteins in *Escherichia coli *(*E. coli*) is a common event. Because of the belief that IBs are intracellular deposits of misfolded, biologically inactive proteins, they have been considered to be a bottleneck in protein production. To avoid time consuming and often unfavorable renaturation procedures, a lot of effort has been made to produce soluble proteins in bacteria either by altering the production process [1,2], by co-expression of chaperones [3,4] or by altering the target protein (point mutations, fusion proteins) [5,6]. In the last few years, the view of inclusion bodies has been changing. The first observations of enzymatic activity in proteins inside IBs [7,8] were reported more than 15 years ago, but the phenomenon was not further explored and the usefulness and biotechnological importance were not recognized. Recently, several groups have reported the presence of biologically active proteins inside IBs [9-12]. The presence of residual native-like structures detected by Fourier transform infrared (FT-IR) spectroscopy has been reported by several authors [13,14].

Previous studies in our laboratory revealed an interesting type of inclusion bodies containing a high percentage of correctly folded protein precursor trapped inside (non-classical IBs – ncIBs). For fermenter cultivation, Jevsevar at al. [12] reported more than 40% activity of granulocyte colony stimulating factor (G-CSF) extracted from ncIBs under non-denaturing conditions, whereas in the shake flask culture the percentage of biologically active G-CSF was even higher [11]. Therefore the research was expanded to other structurally unrelated proteins that all form inclusion bodies upon overexpression in *E. coli*. In addition to human granulocyte colony stimulating factor (G-CSF), green fluorescent protein (GFP), N-truncated form of tumor necrosis factor α bearing an N-terminal histidine tag (His7ΔN6TNF-α), and N-truncated form of Lymphotoxin α (ΔN19LT-α) were studied.

As was previously already reported for diverse proteins by several authors [12,15-17] we also demonstrate that the production of IBs at low temperature results in a higher amount of correctly folded proteins trapped inside IBs. This work is aimed at exploring whether the amount of correctly folded protein trapped in IBs is protein specific. Increased solubility in low concentration detergents was also studied. Solubility of inclusion bodies in mild detergents under non-denaturing conditions enables the extraction of the target protein in biologically active form without any denaturation and renaturation process. This is an interesting, and for downstream processes very important, property.

## Methods

### Strains and plasmids

Recombinant *E. coli *production strain BL21(DE3) (Novagen) was used in this study along with expression plasmids pET3a, pET19b (Novagen), and pQT2 (Qiagen) coding four structurally different proteins.

Details about the production of the BL21(DE3) [pET3a/P-Fopt5] strain for G-CSF production were described by Jevsevar et al. [12].

Human lymphotoxin alpha (LT α) from plasmid BBG 5 (R&D Systems) was amplified and truncated by PCR. The truncated form of LT α (lacking the first 19 amino acids: M^1^LPGVGLTPSAAQTARQHPK^20^) was subcloned into the pET19b plasmid (Novagen) between the NdeI and BspHI restriction sites. Plasmid pET19b-dN19 LT-α was then transformed into the *E. coli *BL21(DE3) production strain.

Synthetic human tumor necrosis factor alpha (TNF α) with *E. coli *optimized codons was obtained from British Biotechnology. The N-terminus was truncated (deletion of the first six amino acids: V^1^RSSSR^6^) and amplified by PCR. The deleted protein was than subcloned into the pQE-TAGZyme-2 plasmid (pQT2 – Qiagen), which encodes for the His 7 affinity tag, between the restriction sites SphI and HindIII [18]. Plasmid pQT2-dN6 TNFα was then transformed into the *E. coli *BL21(DE3) production strain.

Green fluorescent protein (GFP) from plasmid pGFP (Clontech) was amplified by PCR and subcloned into the pET19b plasmid between the restriction sites XhoI and NcoI. Plasmid pET19b-GFP was then transformed into the *E. coli *BL21(DE3) production strain.

### Medium

LBG/amp 100 medium: 10 g/l BBL Phytone Peptone (Becton Dickinson), 5 g/l Bacto Yeast extract (Becton Dickinson), 10 g/l NaCl (Sigma), 100 mg/l ampicillin (Sigma), and 2.5 g/l glucose (Sigma).

GYSP/amp 100 medium: 20 g/l BBL Phytone Peptone (Becton Dickinson), 5 g/l Bacto Yeast extract (Becton Dickinson), 10 g/l NaCl (Sigma), 10 g/l glucose (Sigma), trace elements (FeSO_4_7H_2_O (40 mg/l), CaCl_2_2H_2_O (40 mg/l), MnSO_4_nH_2_O (10 mg/l), AlCl_3_6H_2_O (10 mg/l), CoCl_2_6H_2_O (4 mg/l), ZnSO_4_7H_2_O (2 mg/l), NMoO_4_2H_2_O (2 mg/l), CuSO_4_5H_2_O (1 mg/l), H_3_BO_3_(0.5 mg/l)), and 100 mg/l ampicillin (Sigma).

Inducer: 0.4 mM IPTG (Sigma).

### Culture conditions

Initial bacterial inoculum was prepared in a shake flask culture and grown overnight at 25°C at 160 rpm on the LBG/amp 100 medium. It was then transferred to the GYSP/amp 100 medium and induced with IPTG (immediate induction). The shake flask cultures were incubated at 160 rpm and 25°C, 37°C, and 42°C independently (Kühner linear shaker) until the culture reached the stationary phase. After production, the biomass was aliquoted, centrifuged, and the supernatant was discarded. The bacterial pellet was stored for further analysis.

### Isolation of inclusion bodies

The bacterial pellet was resuspended in 50 mM Tris/HCl, containing 30 mM NaCl. The high-pressure homogenizer Emulsiflex^® ^– C5 (Avestin) was used for cell disruption (operating pressure 75 – 100 MPa); several passages were made. The homogenate was centrifuged at 10,000 g. The supernatant (soluble protein fraction; SN1) was stored for analysis. The pellet of IBs (P1) was washed twice with pure water and used for further analysis.

### Distribution of target protein between cytoplasm and IBs

The bacterial pellet, separated cell fractions after cell disruption (SN1, P1), and samples gathered after extraction of target protein from IBs (SN2, P2) were analyzed by SDS-PAGE. Gels were analyzed densitometrically with a ProExpress Imaging System (Perkin Elmer).

### Infrared analysis

The proteins were prepared in a solution, as highly hydrated ammonium sulfate precipitate and as IBs prepared at three various growth temperatures (25, 37, 42°C). Washed IBs, as well as ammonium sulfate precipitates, were dried in a Savant DNA Speed-Vac system for one-hour prior to analysis to reduce water interference in the infrared spectra. The infrared spectra of protein samples were recorded on a Bruker IFS66S spectrometer equipped with liquid nitrogen cooled MTC detector and Golden-Gate ATR diamond cell. The infrared spectra were used mainly for monitoring the deviations in structures of proteins prepared at different temperatures with respect to properly folded ones. Therefore, the effects of reflection were neglected and the ATR spectra were used as recorded with no additional corrections. Typically, 64 scans were averaged at a resolution of 4 cm^-1 ^and apodized with a triangular function. Solvent spectra were recorded and subtracted before the examination of the amide I bands. The structures of the amide I and amide II regions were analyzed by second derivatives and Fourier deconvolution. The derivative spectra were smoothed by the Savitzky_Golay algorithm applying the 3^rd ^polynomial and 15 smoothing points and normalized on the Tyrosine band near 1517 cm^-1^. The spectra were processed applying the Grams software package.

### Extractability of inclusion bodies

The wet IBs pellet (P1) was resuspended with solubilizing buffer (40 mM Tris/HCl with 0.2% N-lauroyl sarcosine, pH 8.0) at a ratio of 1:40. The suspension was shaken for 24 hours at 20°C and centrifuged at 4400 g for 15 minutes. The supernatant (solubilized target protein from IBs; SN2) and pellet (insoluble fraction of IBs; P2), as well as the supernatant SN1 and pellet P1 (chapter 2.2 Preparation of washed inclusion bodies) were analyzed by SDS-PAGE. The target protein was determined densitometrically as a fraction of the total proteins by profile analysis using a BIO-RAD imaging densitometer – model GS-670. Supernatants were further used for biological activity measurements.

### Biological activity

The biological activity of G-CSF was measured via proliferation activity on the murine myeloblastic NFS-60 cell line [19].

The biological activity of TNF α and LT α was measured via the specific cytotoxic activity on the mouse fibroblast L929 cell line [20,21].

The biological activity of all proteins was measured in two distinctive fractions: in the supernatant (soluble protein produced in the cytoplasm; SN1) and in IBs solubilized with 0.2% N-lauroyl sarcosine (SN2). All the samples were filtered through 0.22 μm centrifuge cellulose acetate filters (Costar SPIN-X™) before testing. Average specific activity of the proteins inside the cell fraction is presented as the result.

### Fluorescence

In the case of GFP, fluorescence was taken as a sign of a correctly folded and oxidized protein. The fluorescence spectrum of GFP soluble in the cytoplasm (SN1) as well as of GFP extracted from IBs (SN2) was compared to the spectrum of an in-house GFP standard isolated and purified in our laboratory.

The fluorescence of soluble GFP and GFP extracted from IBs with 0.2% N-lauroyl sarcosine (SN2) was measured on a QuantaMaster C-61 Spectrofluorometer (Photone Technology International).

## Results and Discussion

### Distribution of target protein between cytoplasm and IBs

The protein yield of all four proteins, G-CSF, GFP, His7ΔN6TNF-α, and ΔN19LT-α, was relatively high, as target proteins represent 35–45% of total cell proteins (Table 1). All four proteins form inclusion bodies, but the distribution of the target protein between the soluble form in the cytoplasm and the aggregated form of IBs depends on the protein.

**Table 1:** Protein yield of target proteins inside bacterial cells is presented, as well as the distribution of target protein between soluble and insoluble cell fraction.

Target protein	% of target protein in total proteins (protein yield)	% of protein in insoluble fraction (IBs) with respect to total target protein
G-CSF	38	98
GFP	45	77
His7dN6TNF-α	39	40
dN19LT-α	35	91

Many environmental factors influence the formation of IBs but predicting whether the target protein will accumulate as a soluble or insoluble fraction in the cell is still almost impossible, since this is dependent on the polypeptide and its sequence [22]. Even changing only one amino acid in the polypeptide sequence can drive a formerly soluble protein to aggregate in the IBs [23].

In the case of G-CSF, almost all protein is found in IBs. As already reported, the amount of correctly folded protein inside the IBs strongly depends on the growth temperature [12], with growth at a low temperature of 25°C resulting into the highest percentage of correctly folded protein.

In contrast, at low growth temperatures, fluorescent GFP (correctly folded and oxidized) was found to be soluble to a large extent in the *E. coli *cytoplasm (data not shown). However, using a bacterial host with a strong promoter, it was possible to force the formation of fluorescent IBs.

Although native trimeric TNF-α is produced well as soluble product in *E. coli *cytoplasm [20], the addition of a histidine affinity tag to the truncated form of TNF-α (His7ΔN6 TNF-α) causes some formation of IBs. However, the majority of the protein still remains soluble in cytoplasm.

On the other hand native LT-α, also known as TNF-β, is a trimeric cytokine very similar to TNF-α, but appears in both soluble and IBs form (data not shown), while the truncated form (dN19 LT-α) occurs predominantly as insoluble IBs (Table 1).

### Infrared studies

The secondary structures of the proteins inside IBs were examined by infrared spectroscopy. The frequency and the shape of the amide I bands in the spectral region between 1690 and 1620 cm^-1 ^are sensitive to different types of protein secondary structure [24]. A band analysis of the amide I region, retrieves accurate information about protein secondary structure [25]. However, the components of the amide I band, which belong to different secondary structure elements, are strongly overlapped. To extract these components, second derivatives and Fourier deconvolution were applied. Both mathematical approaches give an almost identical intrinsic band structure in the amide I region, so solely the results of the second derivates are presented.

The presence of a large amount of correctly folded hG-CSF protein inside IBs prepared at low temperature was confirmed by infrared spectroscopy [12]. At higher temperatures (37°C, 42°C), the infrared spectra of IBs revealed red shifts of the maxima and different shapes pointing to different structures and intermolecular associations of proteins inside the IBs.

Although the protein folding of G-CSF is completely different from GFP, His7ΔN6TNF-α or ΔN19LT-α, even for these proteins the highest amount of correctly folded structure inside the IBs was found when the bacterium growth was performed at relatively low temperatures. Infrared spectra of highly purified GFP and His7ΔN6TNF-α protein solutions, as well as of ammonium sulfate precipitates (ASP) of the pure proteins, were recorded and compared to the infrared spectra of isolated IBs prepared at different cultivation temperatures. ASP represents the correctly folded solid form of the pure protein as was proven by biological activity measurements after precipitate dissolution [12].

**Figure 1:**
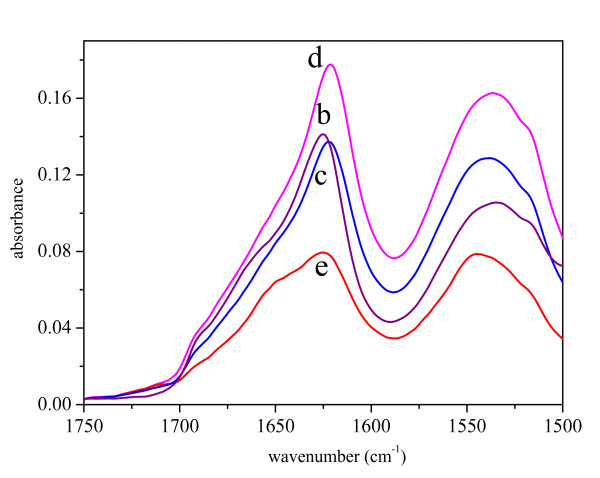
FT-IR spectra in Amide I and Amide II regions of ammonium sulfate precipitate of pure GFP (b), and inclusion bodies isolated from *E. coli *incubated at different temperatures: 25°C (c), 37°C (d) and 42°C (e).

**Figure 2:**
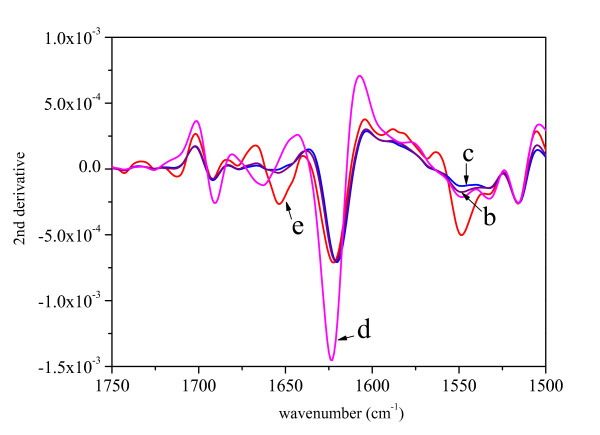
The second derivatives of the spectra presented in Figure 1.

As pure GFP in solution tends to aggregate at high concentrations, the IBs samples were compared to the ASP of pure GFP (Figure 1). The second derivative spectra in the amide I and amide II regions are presented in Figure 2. The distinctive bands characteristic for β-structure [26,27] can be found at 1627 cm^-1 ^and 1686 cm^-1^. The original, as well as the secondary derivative spectra of IBs prepared at 25°C and 37°C are very similar to the spectrum of the pure protein. In contrast, the spectrum of the IBs prepared at 42°C possesses several differences. The most pronounced one is the decrease in the intensity of the band at 1627 cm^-1 ^while the intensity of the band at 1650 cm^-1^, characteristic for unordered protein structure [26], increases.

**Figure 3:**
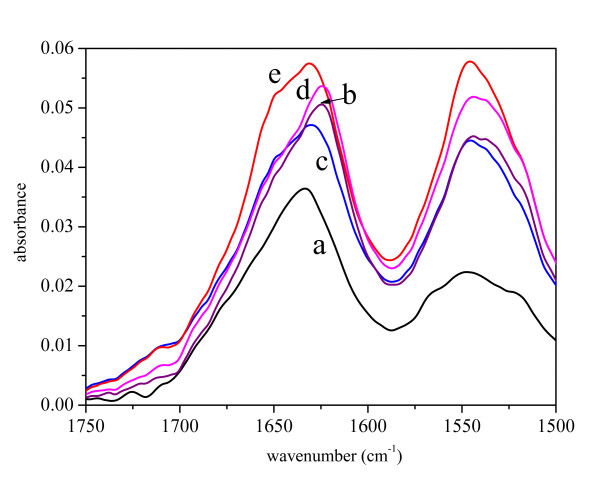
FT-IR spectra in Amide I and Amide II regions of His7ΔN6 TNF-α in solution (a), as ammonium sulfate precipitate (b), and in inclusion bodies isolated from *E. coli *incubated at different temperatures: 25°C (c), 37°C (d) and 42°C (e).

**Figure 4:**
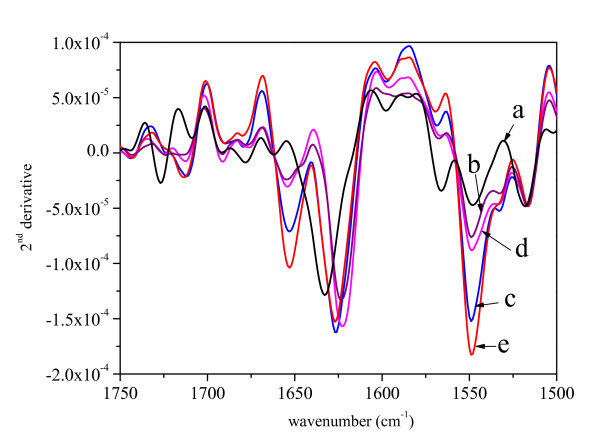
The second derivatives of the spectra presented in Figure 3.

The structure and frequencies of band components of the amide I region in the infrared spectrum of pure His7ΔN6 TNF-α confirmed the predominantly β structure of the protein (Figure 3 and Figure 4). The bands of the ASP of the pure protein moves slightly towards the 1620 cm^-1 ^characteristic for intermolecular β-sheet connections [28]. Upon resuspension, the ASP biological activity of the protein remains unchanged (data not shown), thus no change in the protein structure is expected to occur in ASP. IBs prepared at 37°C show the same spectrum as the ASP, while the spectra of the IBs prepared at 25°C and 42°C have an additional band at 1650 cm^-1 ^characteristic for unordered protein structure [26].

**Figure 5:**
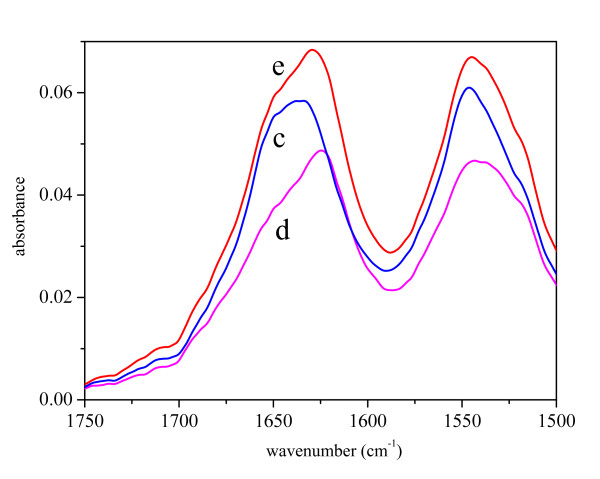
FT-IR spectra in Amide I and Amide II regions of ΔN19 LT-α inclusion bodies isolated from *E. coli *incubated at different temperatures: 25°C (c), 37°C (d) and 42°C (e).

**Figure 6:**
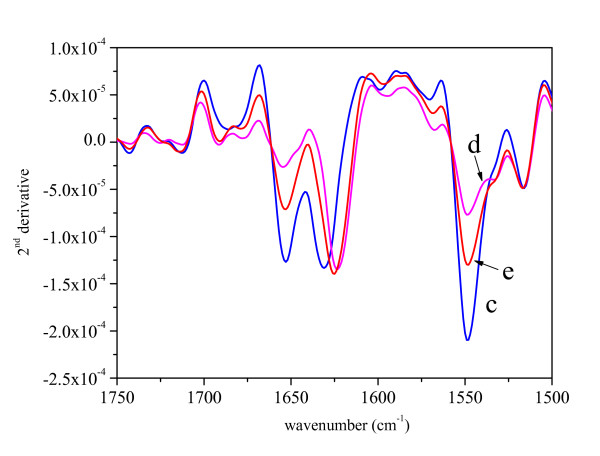
The second derivatives of the spectra presented in Figure 5.

For ΔN19LT-α, only spectra of IBs prepared at different temperatures were recorded since the protein is very hydrophobic and aggregates at high concentrations. ΔN19LT-α has a predominant β-structure that can be seen from the original (Figure 5) and second derivative spectra (Figure 6) of IBs displaying peaks at 1627 cm^-1 ^and 1685 cm^-1 ^[26,27]. The infrared spectra of IBs prepared at different growth temperatures differ from each other. Especially prominent is the increase in the intensity of the band at 1650 cm^-1 ^caused by the elevation of the preparation temperature. This change in band intensity implies an increase in the unordered structure inside IBs with respect to those prepared at lower temperatures

### Extractability of proteins from IBs

**Figure 7:**
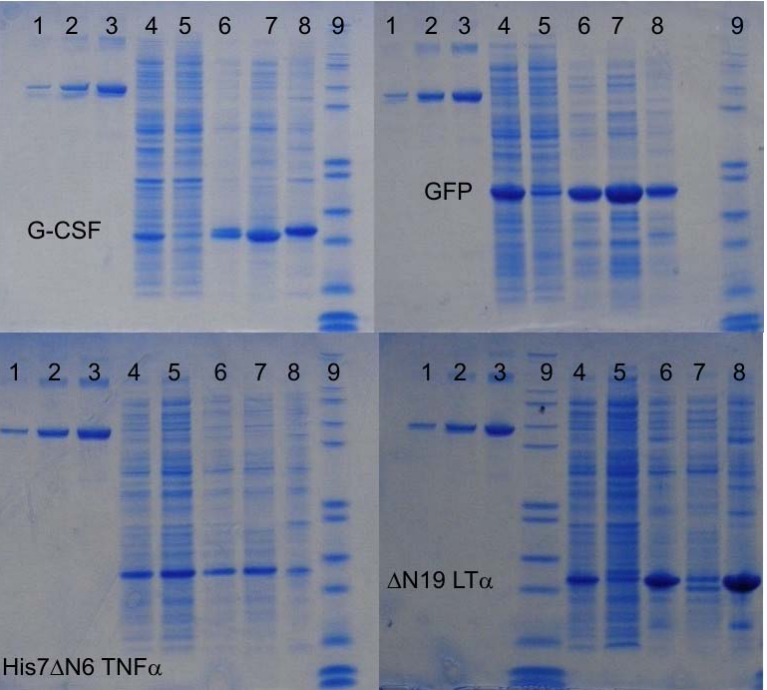
**SDS-PAGE analysis of proteins extracted from IBs with 0.2% N-lauroyl sarcosine**. Inclusion bodies were isolated from *E. coli *cultivated at 25°C. 1 – BSA (Bovine serum albumin) standard 0.2 mg; 2 – BSA standard 0.88 mg; 3 – BSA standard 1.5 mg; 4 – total cell proteins; 5 – soluble (cytoplasmic) proteins; 6 – IBs proteins; 7 – IBs proteins extracted with 0.2% NLS; 8 – in 0.2% NLS insoluble IBs proteins; 9 – LMW standard.

As already described by Jevsevar [12] and Peternel [11,29], G-CSF IBs prepared at 25°C are readily soluble in 0.2% N-lauroyl sarcosine with almost 97% of G-CSF being dissolved. Slightly less protein can be extracted from GFP IBs and His7dN6 TNF-α IBs (80 – 85%) (Figure 7). These proteins, easily extractable from ncIBs, contain a higher percentage of correctly folded protein inside IBs (based on spectroscopy analysis). This supports the hypothesis suggested by Jevsevar at. al. [12] that the more alike the inner structure of the IBs are the easier it is to extract the correctly folded protein from IBs.

On the other hand, the extractability of ΔN19LT-α from IBs with 0.2% N-lauroyl sarcosine is significantly lower, and only up to 40% of the target protein can be dissolved (Figure 7). As can be seen from the infrared studies, the percentage of unordered structures inside ΔN19LT-α IBs is very high (even those formed at 25°C) and this could explain the poor extractability of ΔN19LT-α from such IBs.

In all cases, IBs prepared at higher temperatures (37°C, 42°C) are less soluble in 0.2% N-lauroyl sarcosine. The lower solubility of IBs prepared at higher temperatures is not surprising as IBs have been generally reported to be insoluble in mild detergents. This is probably due to the high percentage of unordered protein structures in the IBs that was also confirmed by spectroscopy analysis.

Extractability of properly folded proteins from IBs in mild detergents is a very interesting property. IBs are predominantly composed of the target protein. During protein extraction in mild, non-denaturing conditions mainly properly folded proteins are extracted. We might thus speculate that during such extraction the target protein is further enriched and therefore less downstream steps are necessary to purify the protein. This hypothesis was confirmed during the preparation of the process for purification of G-CSF, where, in a single chromatography step, the production of a biologically active G-CSF with a purity greater than 95% was achieved [30]. The same was also achieved for the isolation of pure, biologically active, fluorescent GFP and His7dN6 TNF-α (unpublished results).

Here some interesting questions arise, such as whether the IBs of other proteins produced at low temperatures are also in the form of more soluble material containing easily extractable, correctly folded proteins. Since IBs usually represent a highly concentrated pool of the target protein, it is worth exploring the industrial potential of easily soluble IBs, especially those containing large amounts of biologically active protein, in order to reduce production costs and to develop environmentally less harmful technologies. Such IBs composed of pure protein could also be used as active nanoparticles in diagnostics or as biocatalysts in enzymatic processes or even as biopharmaceuticals.

### Biological activity

The specific biological activity of the protein was measured in two distinctive fractions: soluble protein produced in the cytoplasm (SN1) and insoluble protein, solubilized from IBs under mild non-denaturing conditions in 0.2% N-lauroyl sarcosine (SN2). Biological activity was measured for all four studied proteins.

**Figure 8:**
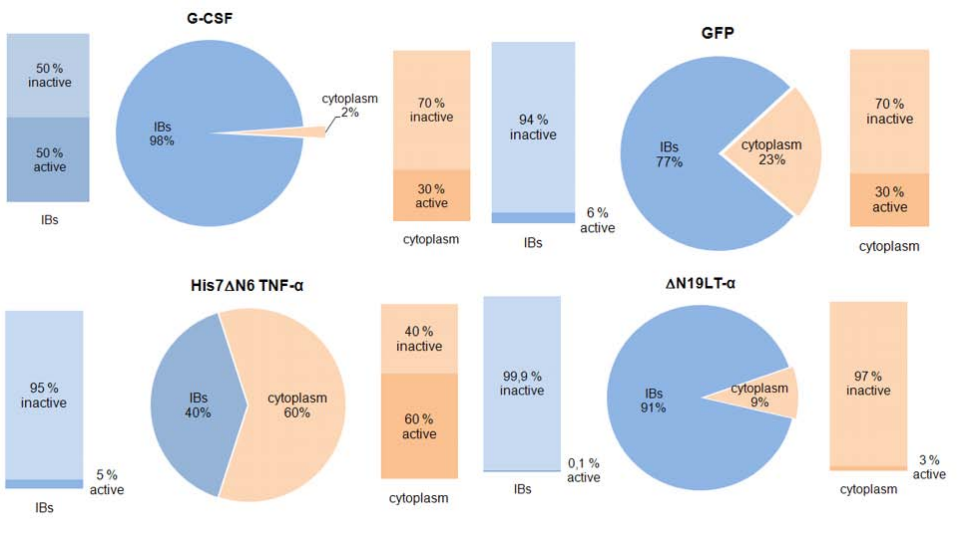
Distribution of target protein between the soluble fraction (cytoplasm) and insoluble fraction (IBs) is presented on pie diagrams. Percentages of specific biological activity of target proteins in each fraction are presented on column charts.

Almost 50% of G-CSF extracted from the IBs shows specific biological activity [11]. Since the majority of the expressed G-CSF is in the form of IBs (98%), this represents almost all the biologically active protein produced in the cell. From the remaining 2% of total GCSF, which is produced in the cytoplasm, only 30% is in active form, representing less than 1% of the total G-CSF (Table 2, Figure 8).

**Table 2:** Specific biological activity of the target protein regarding the total target protein produced in bacterial cell is presented. The distribution between soluble (cytoplasm) and insoluble (IBs) cell fraction is also presented.

Target protein	Specific biological activity of the target protein produced in bacterial cells	Distribution between soluble and insoluble cell fraction	
		cytoplasm	IBs
G-CSF	49,6	0,6	49
GFP	12	7	5
His7ΔN6 TNF-α	38	36	2
ΔN19LT-α	0,4	0,3	0,1

Altogether around 12% of the total GFP produced in bacterial cells is correctly folded and oxidized (fluorescent). As previously reported by Garcia-Fruitos at al. [10], GFP inside the IBs can be highly fluorescent. Our results show that 6% of all GFP in IBs is in the active, fluorescent form. Although the amount of active, fluorescent GFP in the cytoplasm is higher (about 30%), the protein is predominantly produced in the insoluble fraction (77%). Therefore, the share of the total GFP produced in the cell is almost equally distributed between the soluble and insoluble fraction (Table 2, Figure 8). Based on the spectroscopy analysis, we believe that the amount of correctly folded protein inside IBs is even higher, but not all the correctly folded protein is oxidized. Consequently not all the correctly folded protein is fluorescent.

As in the case of GFP, around 5% of His7ΔN6 TNF-α extracted from IBs is in biologically active form. Since 40% of the protein is produced inside IBs, this represents 2% of the total His7ΔN6 TNF-α produced in *E. coli*. Protein produced in the cytoplasm shows much higher biological activity (around 60%) and this corresponds to 36% of the total His7ΔN6 TNF-α produced in the cell. Therefore we can conclude that correctly folded His7ΔN6 TNF-α is predominantly produced in the soluble fraction (Table 2, Figure 8).

The specific biological activity of total ΔN19LT-α produced in bacterial cells is less than half the percentage of that expected. The protein is mainly produced in the form of IBs inside bacterial cells (91%). Since only 0.1% (Table 2, Figure 8) of the protein extracted from IBs is biologically active, we may conclude that IBs of dN19LT-α, even when produced at 25°C, show typical characteristics of classical inclusion bodies containing essentially denatured and almost completely biologically inactive protein. The results were also confirmed by infrared spectroscopy (Figure 5 and 6), which revealed a high percentage of unordered structure in ΔN19 LT-α IBs produced at low temperature. Some specific biological activity was measured in the soluble protein fraction (3%), but even there the amount of properly folded protein is very small.

Based on the results presented above, the properly folded active protein can be produced inside the soluble (cytoplasm) and insoluble (IBs) cell fraction. The amount of properly folded protein inside individual cell fractions depends very much on the protein.

In the case of G-CSF, the active protein was predominantly produced inside the IBs. In addition, the amount of active protein inside GFP and His7ΔN6 TNF-α IBs was significant and IBs could be used the source for the extraction of active protein. In the case of N19 LT-α, the amount of properly folded protein inside IBs was trivial. Therefore we can conclude that three of the four studied proteins form IBs composed of a significant amount of properly folded biologically active protein. As mentioned in a previous section, such IBs containing active proteins are very interesting for research and medical purposes, as well as for industrial use.

Once again it has to be stressed that specific biological activity data discussed above were obtained for IBs prepared at 25°C. When produced at higher temperatures (37°C, 42°C), all the proteins showed almost no biological activity. The same results were also obtained by infrared spectroscopy, where spectra of IBs produced at low temperatures were very similar to the spectra of the pure proteins, whereas IBs produced at higher temperatures contain a high proportion of unordered structures.

## Conclusion

In all cases studied so far, it was found that various amounts of correctly folded proteins were trapped inside inclusion bodies. The amount of correctly folded proteins and the solubility of the inclusion bodies mainly depend on the nature of the target protein, as well as on the growth conditions, such as the temperature and induction regime. Infrared spectroscopy analysis shows the presence of correctly folded proteins inside IBs. However, it is not possible to predict the biological activity of the target protein from spectroscopy analysis of the secondary protein structure. Results show that even though spectroscopy analysis shows a relatively high proportion of correctly folded proteins inside IBs (in the case of GFP and His7ΔN6 TNF-α at 37°C), very low solubility of IBs in 0.2% N-lauroyl sarcosine as well as almost no biological activity can also be observed.

In our future work, we plan to optimize important parameters for the biosynthesis of proteins in the form of inclusion bodies with the main goal of increasing the accumulation of active protein inside the inclusion bodies. This should enable efficient isolation of the active protein by gentle extraction under non-denaturing conditions or even allow the use of IBs as active nanoparticles.

## Authors' contributions

ŠP designed and performed most of the experiments and prepared the manuscript as well as final data analysis and figures. JG performed infrared studies and contributed the expertise on infrared analysis. VGP and RK were consolidating authors and participated in the manuscript preparation. All authors approved the final manuscript.
